# Studying chromosome biology with single-molecule resolution in *Xenopus laevis* egg extracts

**DOI:** 10.1042/EBC20200026

**Published:** 2021-04-16

**Authors:** George Cameron, Hasan Yardimci

**Affiliations:** Single Molecule Imaging of Genome Duplication and Maintenance Laboratory, The Francis Crick Institute, London NW1 1AT, United Kingdom

**Keywords:** Chromosome organisation, DNA repair, DNA replication, Single-molecule techniques, Xenopus laevis

## Abstract

Cell-free extracts from *Xenopus laevis* eggs are a model system for studying chromosome biology. *Xenopus* egg extracts can be synchronised in different cell cycle stages, making them useful for studying DNA replication, DNA repair and chromosome organisation. Combining single-molecule approaches with egg extracts is an exciting development being used to reveal molecular mechanisms that are difficult to study using conventional approaches. Fluorescence-based single-molecule imaging of surface-tethered DNAs has been used to visualise labelled protein movements on stretched DNA, the dynamics of DNA–protein complexes and extract-dependent structural rearrangement of stained DNA. Force-based single-molecule techniques are an alternative approach to measure mechanics of DNA and proteins. In this essay, the details of these single-molecule techniques, and the insights into chromosome biology they provide, will be discussed.

## Introduction

For cells to remain healthy and properly divide, genome stability and appropriate gene expression must be maintained. To do this, DNA packaged into chromatin is replicated, repaired and transcribed. The complex and essential nature of these reactions complicates their dissection in cells and in reconstituted reactions. Embryos of the African clawed frog, *Xenopus laevis*, have historically been used for studying vertebrate development and the cell cycle, but the number of possible manipulations was improved with the development of cell-free egg extracts [1]. *Xenopus* egg extracts are a powerful system to study DNA metabolism and packaging, and other cell cycle-related events like microtubule dynamics. Large volumes of extract, containing necessary factors for vertebrate DNA replication, repair and chromatin organisation, are harvested by crushing eggs and extracts can be frozen. Usefully, extracts are synchronised and can be induced to progress through the cell cycle, using several preparation methods [2,3]. A variety of single-molecule techniques have been used in combination with *Xenopus* egg extracts, encompassing fluorescence-based methods, to visualise molecules, and force-based methods, to mechanically manipulate molecules. Here an overview of single-molecule approaches used to investigate chromosome biology in *Xenopus* egg extracts will be presented.

## Chromosome organisation in *Xenopus* egg extracts

To produce *Xenopus* egg extracts, ovulation is induced in female frogs and eggs are collected. At this point before fertilisation eggs are arrested in metaphase II of meiosis. After removal of a thick jelly layer surrounding eggs and washing, eggs are crushed by centrifugation in buffer containing Ca^2+^ to mimic fertilisation and harvested cytoplasm enters interphase [4]. A fraction from the first centrifugation yields an extract known as low-speed supernatant (LSS). In the 1980s, Blow and Laskey mixed an LSS-like extract with *Xenopus* sperm chromatin, to trigger chromatin decondensation, nuclei formation and semiconservative DNA replication [2].

**Figure 1:**
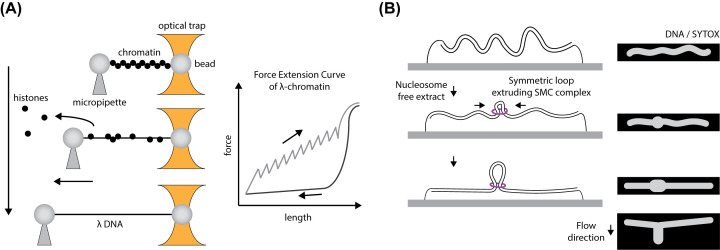
Single-molecule techniques to investigate chromosome organisation (**A**) An optical trap measuring forces involved in chromatinisation of λ DNA in *Xenopus* egg extracts [6]. λ DNA attached to beads at both ends is bound at one end to a micropipette, then incubated in extracts to form chromatin. After chromatin assembly, extract is replaced with buffer and the second bead optically trapped. The micropipette is moved away from the optical trap, and force in DNA fibre is continuously measured, shown here in the force extension curve. (**B**) Adding nucleosome-depleted egg extracts to λ DNA double-tethered to the surface of a coverslip allows loop extrusion to occur. Loops appear as regions of high SYTOX staining on DNA, visualised by TIRF microscopy, unless applying perpendicular flow [15]. Abbreviation: TIRF, total internal reflection fluorescence.

LSS extracts assemble chromatin on naked DNA, providing a system to study mechanisms of chromosome organisation. Before LSS was first used to replicate sperm chromatin, histones in extracts were shown to supercoil circular DNAs [5]. In 2000, this chromatin assembly was visualised at a single-molecule level. Single 48.5 kbp λ DNAs, tethered through biotin–streptavidin interaction to the surface of a glass slide, were stained with fluorescent YOYO dye and visualised with fluorescence microscopy. DNA was stretched under buffer flow in a microfluidic flow chamber, and upon addition of extract, DNA tethered at one end was quickly compacted [6]. Atomic force microscopy (AFM) of chromatinised products showed formation of canonical nucleosome structures, where DNA is wrapped around an octameric complex of two histone H2A-H2B dimers and a H3-H4 tetramer, on λ DNA [6]. λ DNA suspended between two microsphere beads, in an optical tweezers setup, could also be chromatinised in extracts (see Figure 1A). Movement of the micropipette stretches DNA, and discrete steps of DNA unwrapping from individual nucleosomes at forces of 20–40 pN are observed in force extension curves (Figure 1A) [7].

Alongside DNA packaging by histones, other proteins provide higher order chromosome organisation, notably the structural maintenance of chromosomes (SMC) family proteins condensin and cohesin. Both complexes contain two long coiled-coil subunits joined at a hinge region and an ATPase head region, when bound to ATP, forming a structure with a ring topology. A third subunit, kleisin, binds both ATPase heads, and is associated with HEAT-repeat containing regulatory subunits [8,9]. The cohesin complex establishes sister chromatid cohesion during DNA replication and forms DNA loop structures known as topologically associated domains (TADs) on chromosomes during interphase [8,9]. Condensin, on the other hand, organises mitotic chromosomes by forming DNA loops. When antibodies are added to inhibit condensin activity in extracts either during mitotic sperm chromatin condensation, or after mitotic chromosome assembly has been completed, chromosome structure is drastically altered [10]. Remarkably, if nucleosome formation is inhibited in extracts, condensin alone forms chromosome-like structures [11].

A loop extrusion model to explain chromosome condensation by condensin and cohesin has been popularised by single-molecule visualisation of loop extrusion on tethered DNAs with purified proteins [12–14]. Loop extrusion has recently been studied by the Brugués laboratory in extracts, either in metaphase by harvesting eggs crushed without any Ca^2+^ present, or in interphase with LSS-type extracts [15]. Single-molecule total internal reflection fluorescence (TIRF) microscopy was used to visualise surface-tethered λ DNAs stained with fluorescent dye in both cell-cycle stages. TIRF provides a thin layer of illumination at the coverslip surface, giving a good signal-to-noise ratio suitable for visualising individual molecules. When extract is added to DNAs tethered to the surface at both ends nucleosome formation removes slack from DNA and prevents loop formation by SMC complexes. H3-H4 was depleted from metaphase and interphase extracts to prevent nucleosome formation, and in both types of extract DNA clusters were observed to form and increase in size with time. Applying perpendicular flow showed these DNA clusters form a loop topology (Figure 1B). In metaphase extracts, loop extrusion occurs asymmetrically from one side of the loop and does not occur after condensin depletion. Interphase extracts have loop extruding activity, but in this cell-cycle stage extrusion is symmetric and dependent on cohesin complexes. Immunostaining shows condensin co-localisation with metaphase loops and cohesin co-localisation with interphase loops. The physiological context of *Xenopus* egg extracts will be useful in determining how the cell cycle controls loop extrusion by condensin and cohesin complexes.

## Replicating DNA in *Xenopus* egg extracts

Until the recent reconstitution of budding yeast DNA replication with purified proteins [16,17], *Xenopus* egg extracts served as the only *in vitro* system to study eukaryotic DNA replication. LSS replicates *Xenopus* sperm chromatin [2], but this depends on nuclei formation, complicating real-time single-molecule imaging of replicating DNAs. To visualise individual DNA molecules after completion of replication in egg extracts, DNA combing and electron microscopy have been used [18]. In DNA combing, DNA is replicated in extracts with sequential addition of modified nucleotides such as digoxigenin- and biotin-modified deoxyuridine. DNA is stretched on glass slides and immunofluorescence is used to identify positions of modified nucleotides. Early replicating DNA is marked by digoxigenin, whilst later replicating DNA is biotin-labelled. Analysis of these labelled tracts has been used to determine the inter-origin distance, replication fork rates and factors controlling origin firing in *Xenopus* extracts [19,20].

**Figure 2:**
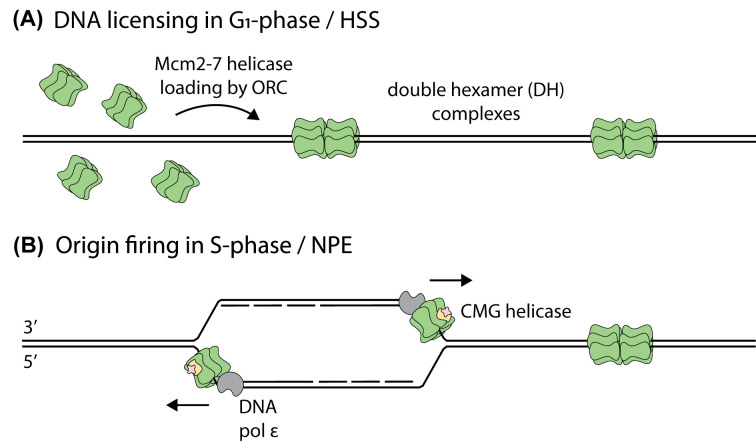
DNA replication in *X. laevis* extracts (**A**) During G_1_-phase, DNA is licensed at origins of replication. The ORC, Cdc6 and Cdt1 load Mcm2-7 helicases as inactive DH complexes around dsDNA. HSS extracts license DNAs with DHs, with ORC in HSS binding DNA in a sequence-independent manner, so specific origins are not required and different DNAs can be licensed in HSS [51]. (**B**) In S-phase, a number of essential firing factors initiate DNA replication. Mcm2-7 from DHs is remodelled into the CMG helicase which binds ssDNA and unwinds DNA in the 3′-to-5′ direction. Other replisome components assemble around CMG [52]. The events of origin firing are recapitulated by adding NPE to DNAs licensed in HSS.

A system to replicate DNA *in vitro* without nuclear assembly, using two sets of extracts, was developed by Walter, Sun and Newport in 1998 [3,21]. The first extract, high-speed supernatant (HSS), is an interphase extract with membranes removed. HSS facilitates loading of Mcm2-7 helicases on to DNA during DNA licensing, at this point as inactive double hexamer (DH) complexes (Figure 2A). Loading of Mcm2-7 in *Xenopus* extracts is dependent on the origin recognition complex (ORC), Cdc6 and Cdt1, whilst independent of DNA sequence. Nucleoplasmic extract (NPE) is made by adding sperm chromatin to LSS and harvesting the nuclei formed. NPE is mixed with licensed DNA to initiate DNA replication, and Mcm2-7 is activated through remodelling into the Cdc45–Mcm2-7–GINS (CMG) helicase, which unwinds DNA (Figure 2B). Numerous additional proteins present in extracts assemble the replisome complex around CMG. Once licensed in HSS, diverse DNA substrates undergo a single complete round of DNA replication in NPE as origin licensing and firing are temporally separated.

## TIRF microscopy to visualise DNA replication in microfluidic flow cells

**Figure 3:**
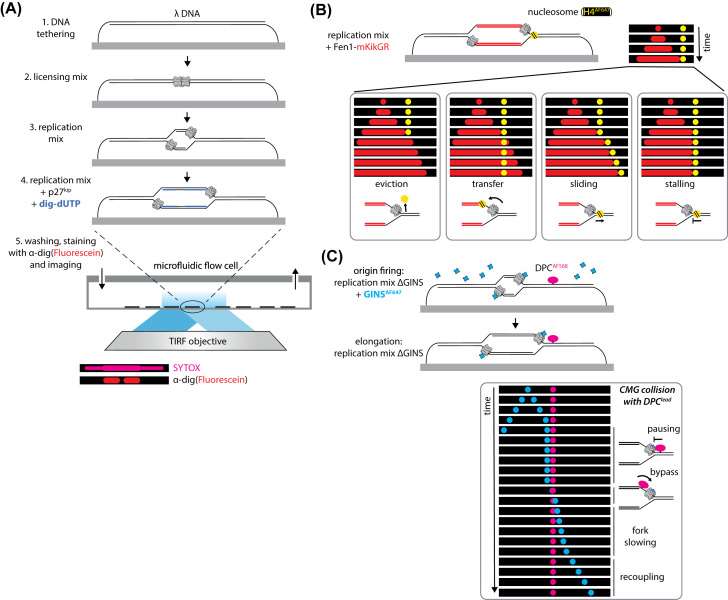
Single-molecule techniques to study DNA replication in extracts (**A**) λ DNA can be replicated in a microfluidic flow cell using a licensing mix, containing HSS, followed by a replication mix, containing HSS and NPE. Adding a second replication mix containing the CDK inhibitor p27^kip^ prevents excessive origin firing. Digoxigenin-dUTP labels nascent DNA, which can be immunostained [22]. (**B**) Real-time imaging of replication forks in extracts with Fen1-mKikGR, which binds nascent DNA [25], and adaptation of this system to visualise the fate of labelled nucleosomes reconstituted on λ DNA [26]. The right hand panel shows a kymogram, a stack of images from a single position of interest, with a growing replication bubble marked by Fen1-mKikGR. After the right hand replication fork reaches the labelled nucleosome four different histone fates are observed. (**C**) Summary of the KERHMIT assay developed in [33]. The bottom panel shows an example kymogram, where the right hand replication fork collides with a stable leading strand DPC. After initial pausing at the DPC, CMG is shown to bypass the DPC, with the fork rate slowing down until possible recoupling to polymerase. Abbreviation: DPC, DNA–protein cross-link.

An alternative to DNA combing was developed by the van Oijen and Walter laboratories, where surface-tethered DNA is replicated with HSS/NPE inside a microfluidic flow cell, and nascent DNA and associated proteins are visualised after completion of replication (Figure 3A) [22]. After DNA tethering, a licensing mix containing HSS is infused into the flow cell and Mcm2-7 DHs are loaded on to λ DNA. Origin firing begins after addition of a replication mix containing HSS and NPE. This is followed by replication mix with the initiation inhibitor p27^kip^, to prevent firing from multiple origins, and digoxigenin-modified dUTP (dig-dUTP) to label replicated DNA. After finishing replication, fluorescently labelled antibodies stain dig-dUTP and DNA-bound proteins for imaging with TIRF microscopy, correlating protein binding sites with positions of DNA replication.

Visualisation of single DNA molecules using this assay elucidated details of the eukaryotic replication reaction. Similar replication efficiencies for single- and double-tethered DNAs provided evidence that CMG helicases from the same origin do not have to remain physically associated with one another for replication [23]. Modification to the λ DNA template was used to investigate the mechanism of CMG translocation during unwinding [24]. By site-specifically conjugating a quantum dot (QDot) to one strand of λ DNA, then comparing replication fork fate after reaching the QDot from either direction, authors showed replisomes stall frequently when meeting a QDot roadblock on the leading-strand template. Stalling is less common when the replisome reaches a QDot roadblock on the lagging-strand template. This supported a model of CMG translocation on the leading-strand template in a 3′-to-5′ direction.

## Imaging eukaryotic DNA replication in real-time

The replication of tethered DNAs in flow cells with extracts enabled development of real-time single-molecule imaging of DNA replication. A lagging-strand processing enzyme, Fen1, was fused to a photo-switchable fluorescent protein, mKikGR, and included in replication mix. Fen1-mKikGR competes with endogenous Fen1 for binding to nascent DNA and Fen1-mKikGR retention on DNA allows replication fork progression to be imaged in real-time (Figure 3B) [25]. By visualising new initiation events in real-time with Fen1-mKikGR, inter-origin distances were shown to be shorter than previously measured on plasmid or sperm chromatin templates. The examples discussed here show the power of live-imaging in egg extracts for investigating replication fork collisions with proteins on λ DNA.

Real-time imaging of DNA replication with Fen1-mKikGR was adapted to probe dynamics of individual nucleosomes after replication fork collision [26]. Histones and corresponding post-translational modifications must be transferred to daughter DNA strands during replication, but the molecular mechanism of histone inheritance is poorly understood. Individual histone proteins were labelled with fluorophores and reconstituted into nucleosomes on biotinylated λ DNA, appearing as ‘beads-on-a-string’ when λ DNA was double-tethered and imaged with TIRF microscopy. After origin licensing and firing, collisions between replication forks, labelled with Fen1-mKikGR, and individual nucleosomes were observed (Figure 3B). Different events were seen after collision: histone eviction from DNA, histone transfer behind forks, histone sliding ahead of forks, fork stalling or a combination of these events. With labelled H3 or H4, the most common event observed was histone eviction, which was unexpected given the requirement for epigenetic inheritance. A high concentration of free histones in egg extracts is needed to support 12 cell divisions before zygotic transcription begins [27]. High histone concentration might influence the fate of parental histones upon replication fork collision, a hypothesis supported by immunodepletion of H3-H4 from replication mix resulting in histone transfer becoming dominant over eviction. Adding recombinant H3-H4 to depleted extracts restored histone eviction as the dominant event. From this evidence, localised inheritance of histones appears to depend on free histone concentration. In the case of egg extracts, high concentrations of free histones may result in free histones exchanging with parental histones during parental histone transfer to daughter strands, resulting in eviction of parental histones. This has wider implications for how chromatin accessibility may influence nucleosome dynamics during replication [28]. Visualising dynamics of single nucleosomes in egg extracts could be useful for investigating specific roles of replisome-associated factors in nucleosome inheritance.

The mechanism of sister chromatid cohesion establishment can be studied with *Xenopus* egg extracts. Cohesin loading in extracts is dependent on licensing of DNA with Mcm2-7 DH complexes [29]. Single-molecule assays developed to load fluorescently labelled *Xenopus* cohesin on to tethered λ DNAs showed that cohesin dynamically translocates after loading [30]. Cohesin translocation is prevented by addition of the accessory Wapl–Pds5 heterodimer, whilst cohesin phosphorylation or acetylation increases translocation. During DNA replication, cohesin loaded onto parental DNA during interphase must provide cohesion between the new sister chromatids. The fate of cohesin loaded on λ DNA has been followed during DNA replication using replication forks labelled with Fen1-mKikGR [30]. When growing replication bubbles reach labelled cohesin, a number of events were observed, including translocation of cohesin with the moving fork, replication-dependent removal of cohesin and in over 30% of cases incorporation of cohesin into the replicating DNA. Development of this work will further our understanding of cohesion establishment during DNA replication.

Other encounters with DNA-bound proteins, like DNA–protein cross-links (DPCs), during replication are potentially toxic. DPCs caused by common agents [31] are proteolysed after collision by the replisome to allow completion of replication [32]. A single-molecule assay using egg extracts, coined KEHRMIT (kinetics of the eukaryotic helicase by real-time molecular imaging and tracking), used fluorescent CMG to investigate CMG dynamics during DPC repair [33]. The Walter laboratory replaced endogenous GINS complexes in replication mix with labelled recombinant GINS (Figure 3C). Labelled CMG moved at the head of Fen1-mKikGR labelled replication bubbles at comparable speeds to those observed with unlabelled CMG (∼400 nt/min) [25]. After initial characterisation of this system on λ DNA templates, replication was performed on a template containing labelled DPCs. When inhibiting DPC proteolysis, CMG was seen to bypass an intact DPC on the lagging strand (DPC^lag^) following a short pause, consistent with results from ensemble experiments [24,32], but remarkably DPC^lead^ could also be bypassed following a longer pause (Figure 3C). In bulk experiments, DPC^lead^ bypass by CMG depends on the RTEL1 helicase, which acts on the opposite side of the replication fork to CMG, and whose activity is a requirement for efficient DPC^lead^ proteolysis. The authors speculated unwinding by RTEL1 provides ssDNA on the distal side of DPC^lead^, after which CMG can undergo DPC^lead^ bypass either by opening an internal gate in CMG to translocate past DPC^lead^ or by threading unfolded DPC polypeptides through the central channel. After pausing at stable DPC^lead^, CMG was observed in KEHRMIT assays to move markedly slower on DNA (<100 nt/min), possibly reflecting CMG uncoupling from the leading-strand DNA polymerase that remains stalled at the unrepaired DPC^lead^ [34]. In some cases, slowly moving CMG was observed to revert to faster translocation, suggesting recoupling to the polymerase. The KEHRMIT assays discussed here reflect the diverse information gained from using single-molecule techniques, in this case showing CMG dynamics at DPC^lead^ and DPC^lag^, before, during and after collision, in the same experiment.

In new work, single molecule assays with labelled GINS have been used to investigate the signal for CMG removal during DNA replication termination [35]. Timely CMG removal is important to prevent premature CMG unloading and under-replication of DNA. In this work, converging CMGs were visualised, and seen to pass one another if CMG unloading was inhibited. One model suggested CMG unloading after meeting ssDNA–dsDNA junctions, at the lagging strand of a converging replication fork, but the authors suggested this was not the case, as CMGs are still removed when nascent DNA is not present. Taken together with results from bulk experiments an alternative model was proposed where CMG is protected from unloading during elongation by a DNA structure that is lost upon termination. The consequence of CMG meeting ssDNA breaks has also been recently investigated with labelled GINS [36]. After meeting a leading strand nick, CMG falls off the broken end of DNA. In contrast CMG translocates past a lagging strand nick and transitions on to dsDNA, then is removed, likely by the same mechanism as during termination.

## Dissecting the non-homologous end joining mechanism by single-molecule FRET

*Xenopus* egg extracts contain components for vertebrate DNA repair pathways [37] and have been used in single-molecule assays to better understand the non-homologous end joining (NHEJ) mechanism. There are two major vertebrate DNA double-strand break (DSB) repair pathways, homologous recombination (HR) which uses sequence homology between broken strands and undamaged sister chromatids, and NHEJ which directly ligates DSBs [38]. Both LSS and HSS extracts efficiently join different DNA end structures by NHEJ [39]. NHEJ begins after binding of Ku70/80 heterodimer to DSBs, which recruits two enzymes, DNA-dependent protein kinase catalytic subunit (DNA-PKcs) and DNA ligase IV (LIG4), along with the scaffold proteins XRCC4 and XLF [38].

**Figure 4:**
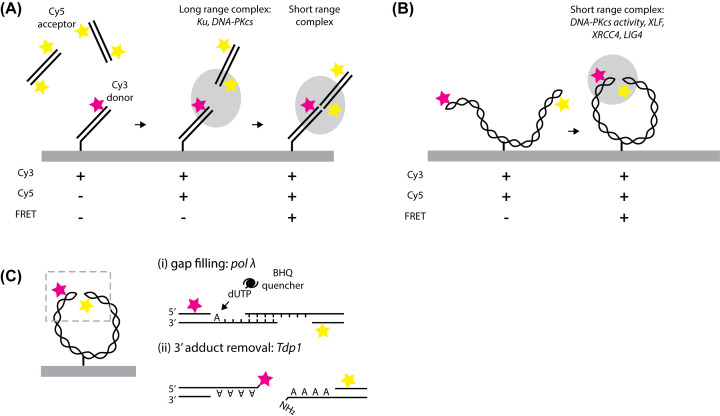
Studying the mechanism of NHEJ by smFRET (**A**) Intermolecular end joining assay for monitoring NHEJ in HSS. The Cy3 donor fluorophore is attached to a 100-bp DNA on the surface of a coverslip inside a microfluidic flow cell. Cy5 association with Cy3 with no FRET shows long range complex formation, followed by conversion into short range complexes, where FRET between the fluorophore pair occurs [40]. (**B**) Intramolecular end joining assay, where Cy3 and Cy5 fluorophores are at the ends of a DNA fragment bound to the surface with an internal biotin [40]. Cy3 and Cy5 continuously are present in a diffraction-limited spot but only show FRET when a short-range complex forms. (**C**) Modifications to intramolecular end joining assays for monitoring enzymatic processing of non-compatible DNA ends [42]. (i) pol λ fills gaps in substrates with resected 3′ DNA ends, detected here by incorporation of a nucleotide modified with BHQ, which quenches Cy3 fluorescence. (ii) Tdp1 removes adducts on 3′ DNA ends, in this example removing Cy3 modification at one 3′ DNA end.

To investigate the order in which protein factors synapse DSBs, single-molecule Förster resonance energy transfer (smFRET) assays were developed in the Walter and Loparo laboratories using HSS extracts [40]. In an intermolecular end-joining assay a 100-bp Cy3-labelled DNA, tethered to the surface of a glass slide, was mixed with HSS and a 100-bp Cy5-labelled DNA (Figure 4A). When Cy3 and Cy5 come within <100 Å of one another they are close enough for FRET, and Cy5 (acceptor) emission occurs during Cy3 (donor) excitation. During intermolecular end-joining, FRET was observed after a lag period, when Cy5-labelled DNA associated with Cy3-labelled DNA without FRET, leading to the proposal that DSB synapsis involves a long-range interaction before short-range complex formation. Long-range complexes were shown to require Ku and DNA-PKcs, but not DNA-PKcs activity. To better study the short-range complex, an intramolecular tethering assay was developed where a linearised DNA, with Cy3 and Cy5 at opposite ends, was tethered to the surface (Figure 4B). DNA-PK catalytic activity was essential for transition to the short-range complex, alongside the XLF, XRCC4 and LIG4 proteins, although a catalytically inactive LIG4 could rescue synapsis without ligation. One previous NHEJ model proposed XLF-XRCC4 forms filaments during NHEJ, however, a modified three-colour intramolecular end-joining experiment including labelled XLF suggested XLF binding as a dimer preceded short-range complex formation [41].

A hurdle that NHEJ must overcome is ligation of non-compatible DNA ends after DSB, for instance non-complementary overhangs or ends with damaged nucleotides. Egg extracts were used to investigate the timing of end processing enzymes making DNA ends compatible for NHEJ [42]. DNA polymerase λ (pol λ) is an example of an end processing enzyme which specifically fills gaps in substrates with resected 3′ ends. Gap filling was shown using bulk assays not to occur if short-range complex formation is inhibited through XLF or XRCC4 depletion or DNA-PKcs inhibition. Intramolecular smFRET assays were adapted to investigate gap filling, through replacement of endogenous nucleotides with a nucleotide mix containing dUTP labelled with a quencher (dUTP-BHQ) (Figure 4C(i)). When dUTP-BHQ was incorporated by pol λ, Cy3 fluorescence was quenched, and consistent with bulk assays quenching occurred mostly after short range complex formation as indicated by FRET. An original smFRET assay for removal of 3′ Cy3 donor adducts by tyrosyl-DNA phosphodiesterase 1 (Tdp1) (Figure 4C(ii)), similarly showed end processing by Tdp1 occurring after short range complex formation. Limiting end processing to the short range complex may be advantageous as compatible ends will undergo ligation without any processing, thus minimising errors [42]. Once incompatible ends are suitably processed, they avoid further processing as they are immediately available for ligation in the short-range complex. SmFRET assays used in this work allowed monitoring of synapsis and end processing together, in real-time, which would be difficult to achieve in bulk assays.

## Conclusions and future outlook

*Xenopus* egg extracts are a powerful *in vitro* system as large volumes of cell-free extract synchronised in the cell cycle can reconstitute complex reactions involving chromosomes. Extending assays in extracts to provide single-molecule resolution gives an ability to follow discrete interactions in a single experiment [33], measure frequencies of heterogenous events [26,30] and measure dynamic structural rearrangements of proteins and DNA [6,15,40]. The use of TIRF microscopy to monitor reactions on surface tethered DNAs has proven particularly valuable.

Control of the cell cycle, with separation of origin licensing and firing in the HSS/NPE system, makes egg extracts useful [3]. Although genetic engineering is not routine, extracts are manipulated through immunodepletion, chemical inhibition and addition of modified substrates. Similar manipulations, that are difficult to perform *in vivo*, can be used in reconstituted eukaryotic systems, like those developed to load cohesin complexes onto DNA [43,44], replicate DNA with purified proteins [16,45–47] or join DNA ends together with purified recombinant NHEJ proteins [48,49]. Individual components of a reconstituted reaction can be added in a stepwise manner, excluded, labelled or mutated without the need for immunodepletion or inhibition. Equally, reconstitution involves purification of multiple proteins and fine-tuning of reaction conditions, whilst extracts contain a complete complement of proteins required for the reactions discussed here, including previously uncharacterised proteins. This justifies development of new single-molecule approaches in extracts to give a detailed insight into pathways like non-NHEJ DNA repair mechanisms and transcription [50]. Further study of the intersection between chromosome replication, repair and organisation in egg extracts will be interesting [26,33].

## Summary

- Extracts from *X. laevis* eggs can be used to study reactions in chromosome biology *in vitro* in combination with single-molecule approaches.
- Surface-tethered DNAs can be replicated with the HSS/NPE system and be visualised in real-time, which has been useful for analysing the consequences of collisions between replication forks and DNA-bound proteins.
- smFRET assays have shown during DSB repair by NHEJ a long-range synaptic complex protects DNA ends before end-processing and ligation in a short-range synaptic complex.
- Extracts can be used for studying DNA organisation into chromatin and higher order structures by SMC complexes. Use of extracts to combine the study of chromatin organisation with other reactions on DNA promises to be exciting.

## Open Access

Open access for this article was enabled by the participation of The Francis Crick Institute in an all-inclusive *Read & Publish* pilot with Portland Press and the Biochemical Society under a transformative agreement with JISC.

## Abbreviations

CMG: Cdc45–Mcm2-7–GINS
DH: double hexamer (of Mcm2-7)
DPC: DNA–protein cross-link
DSB: double-strand break
HSS: high-speed supernatant
KEHRMIT: kinetics of the eukaryotic helicase by real-time molecular imaging and tracking
LSS: low-speed supernatant
NHEJ: non-homologous end joining
NPE: nucleoplasmic extract
QDot: quantum dot
SMC: structural maintenance of chromosomes
smFRET: single-molecule Förster resonance energy transfer
TIRF: total internal reflection fluorescence
